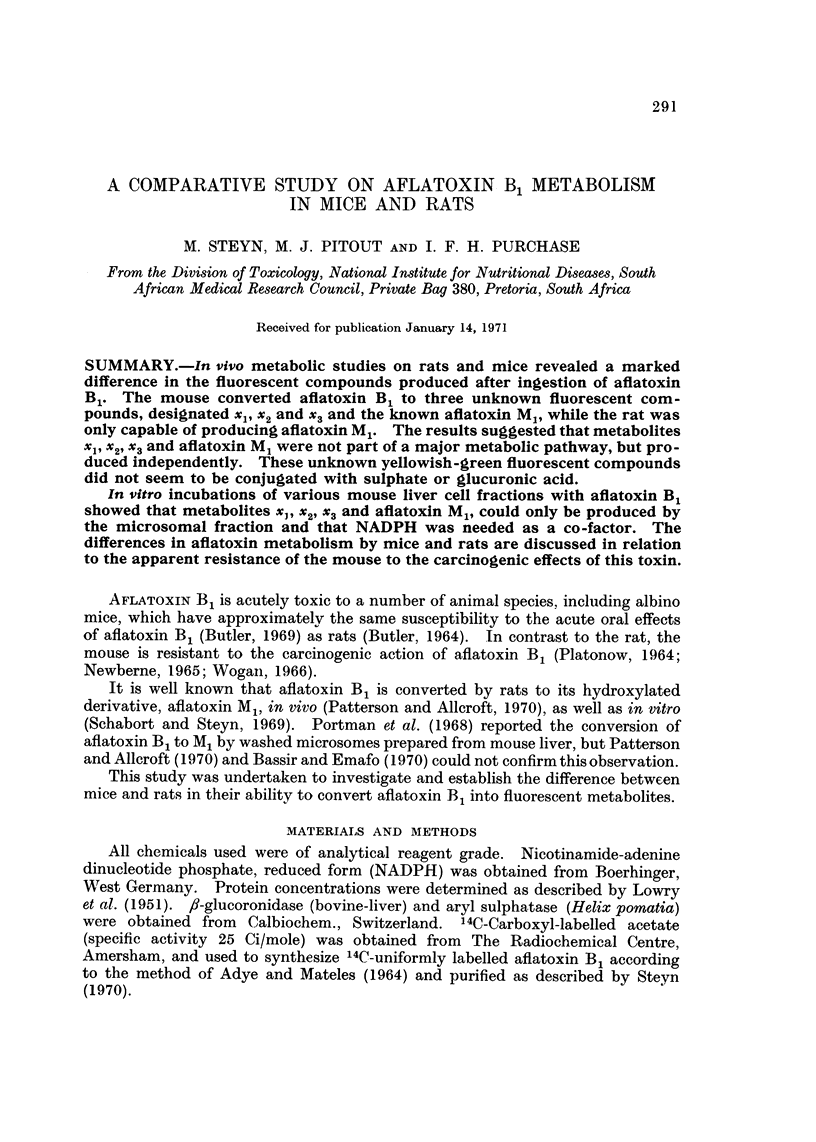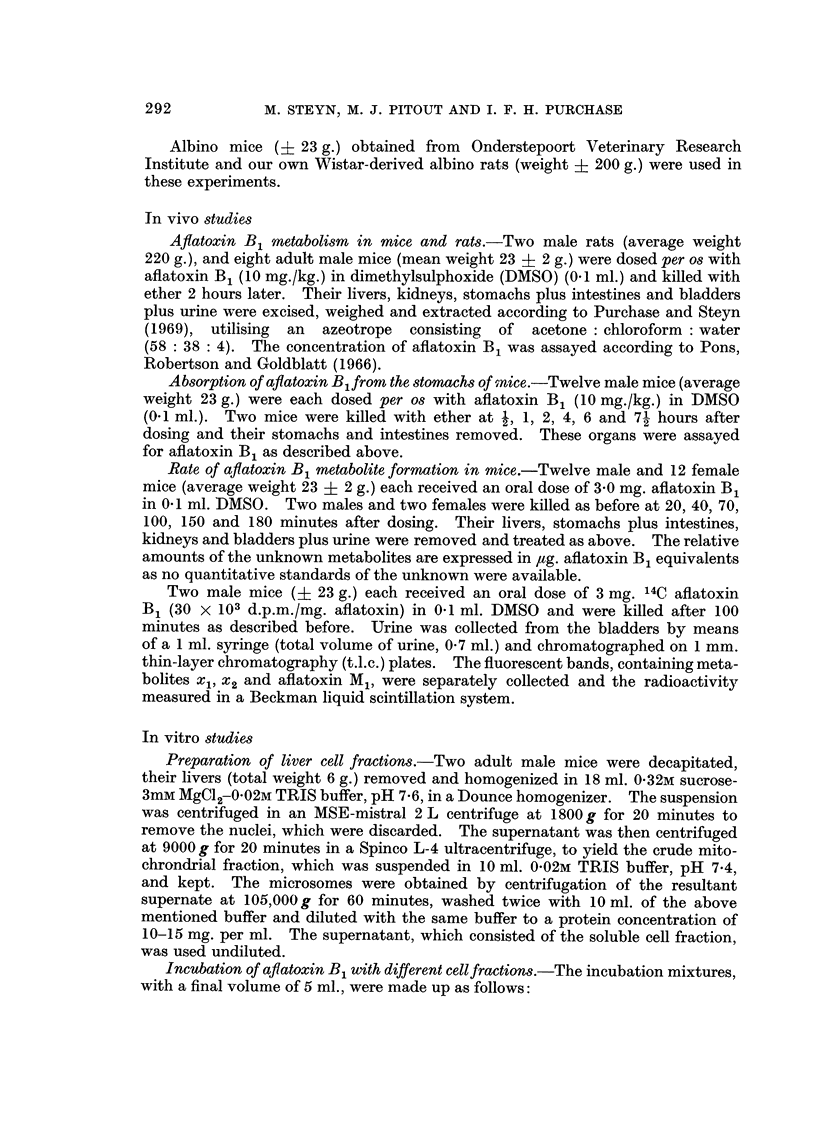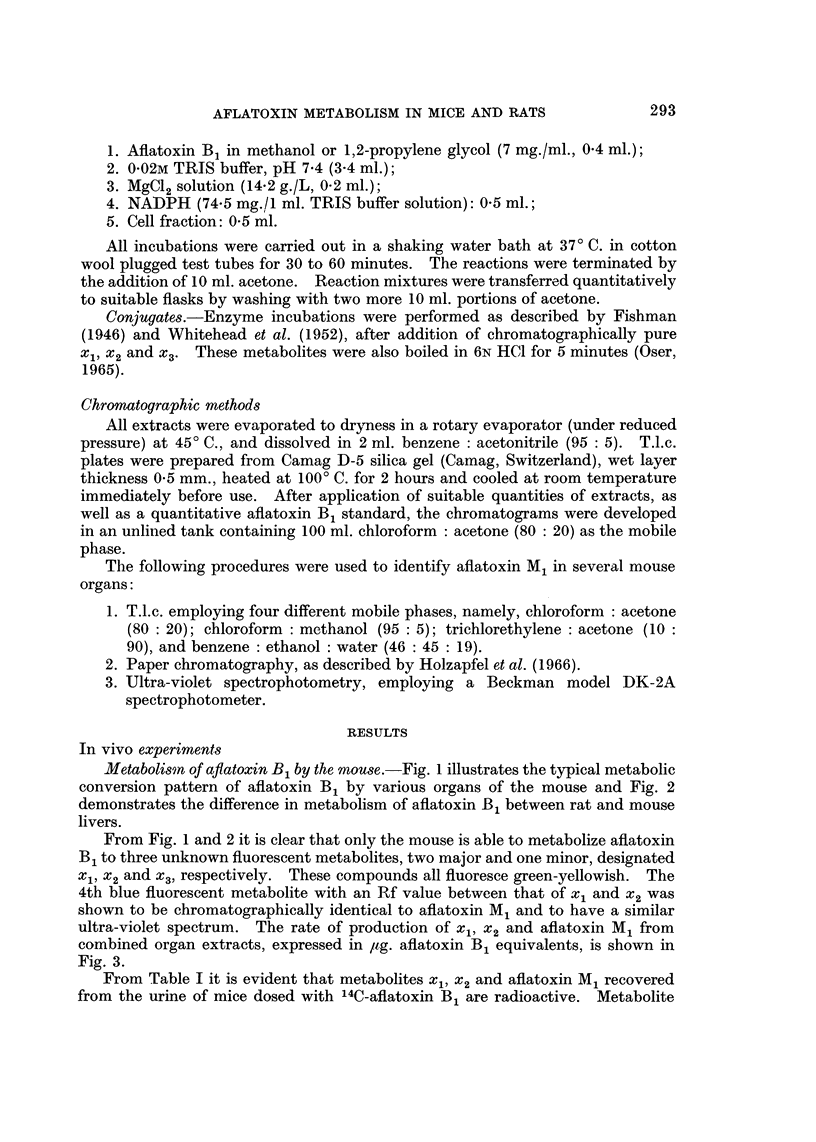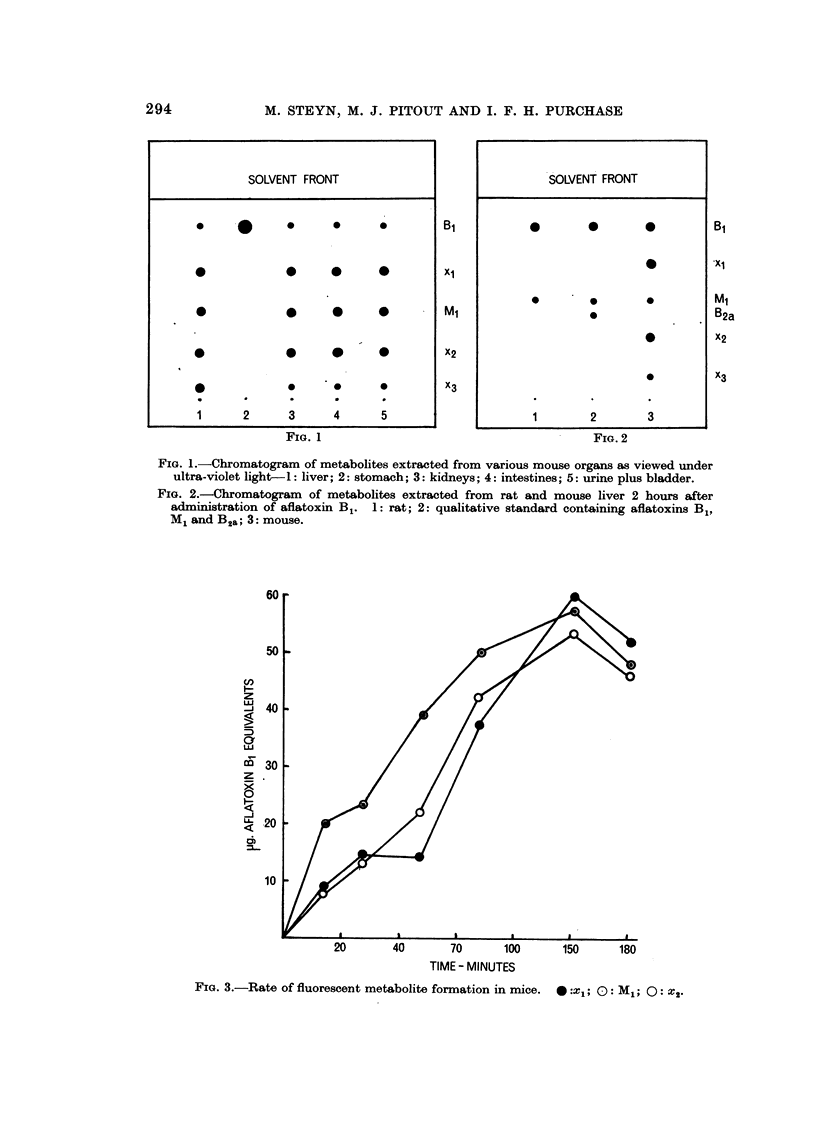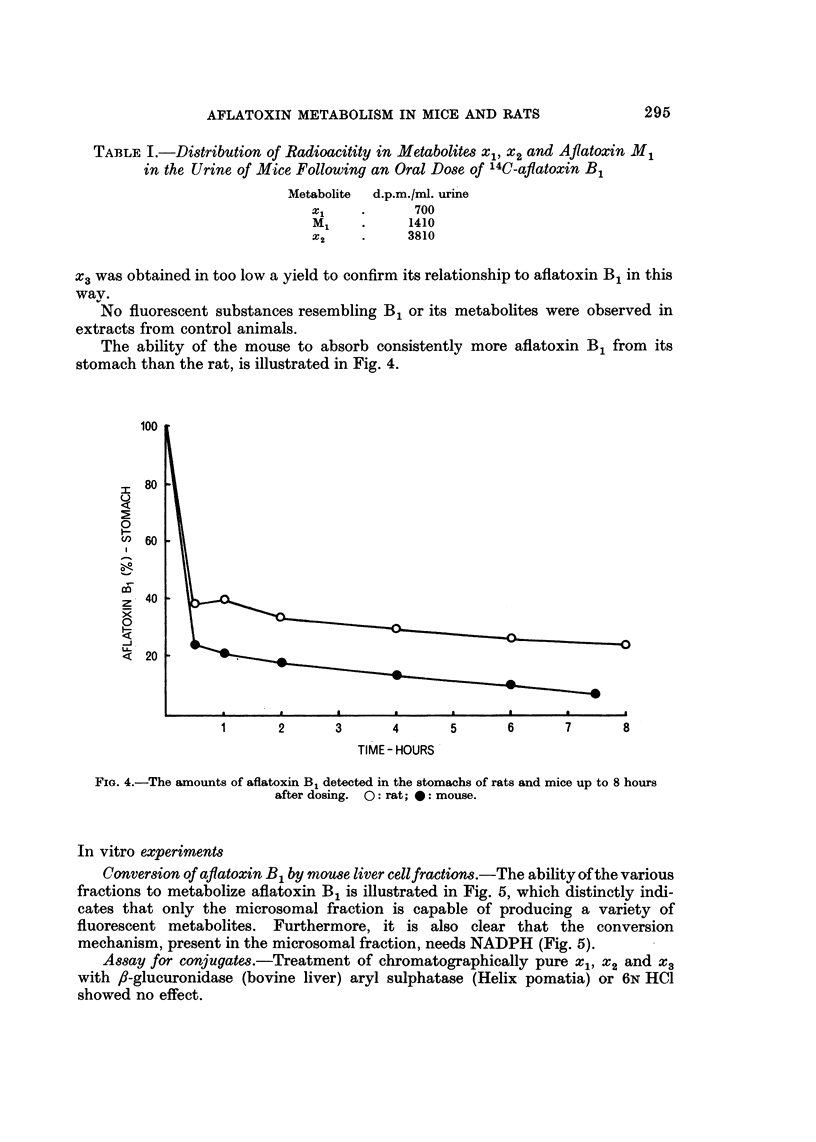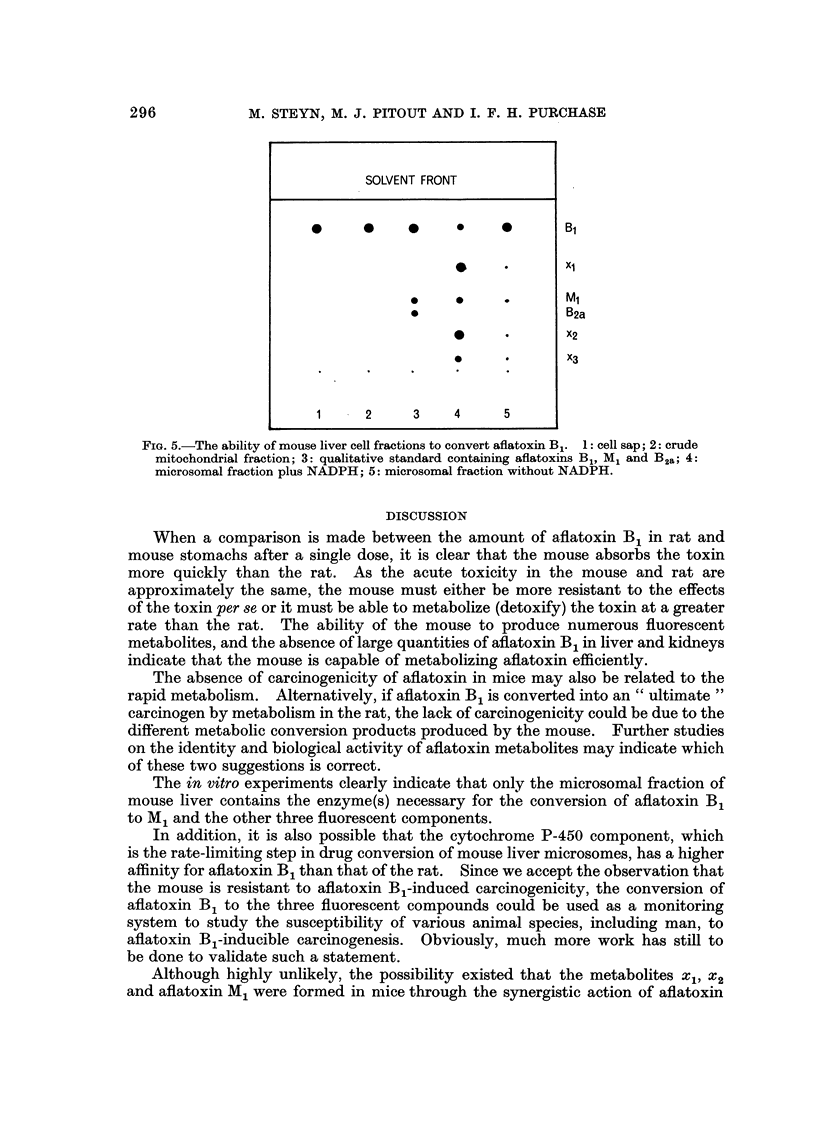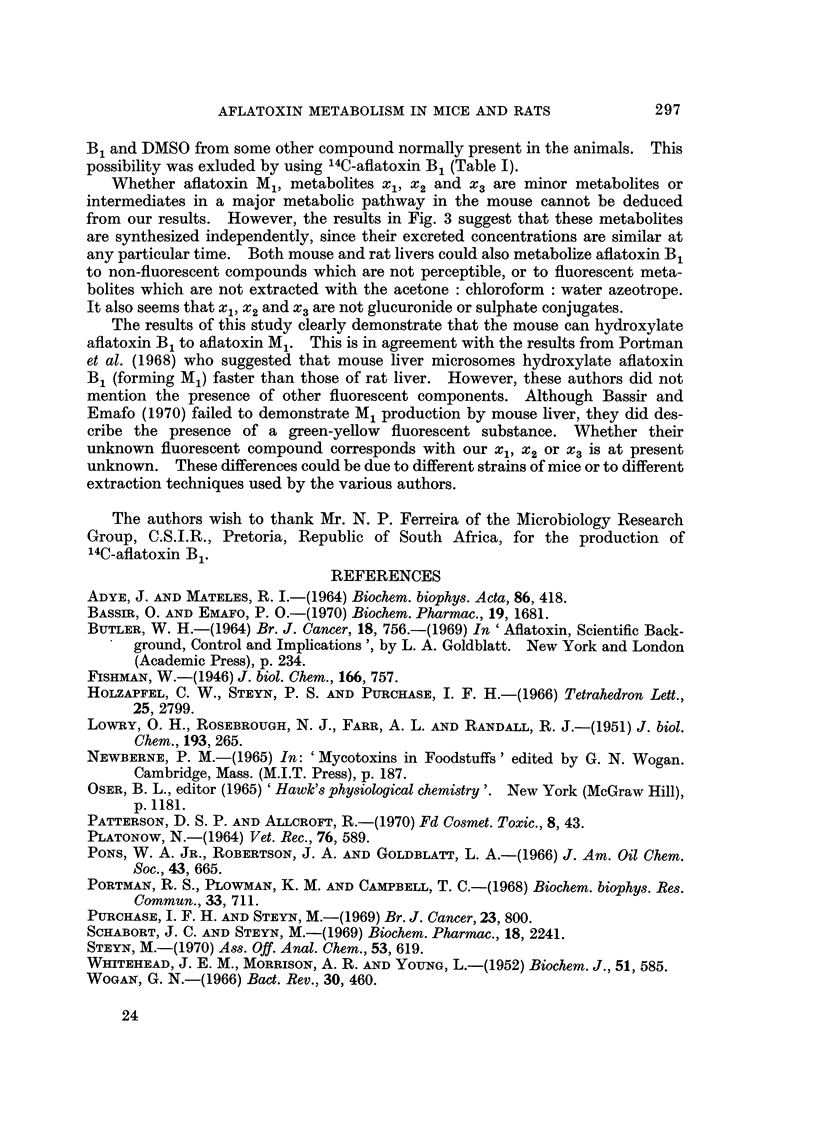# A Comparative Study on Aflatoxin B_1_ Metabolism in Mice and Rats

**DOI:** 10.1038/bjc.1971.37

**Published:** 1971-06

**Authors:** M. Steyn, M. J. Pitout, I. F. H. Purchase

## Abstract

*In vivo* metabolic studies on rats and mice revealed a marked difference in the fluorescent compounds produced after ingestion of aflatoxin B_1_. The mouse converted aflatoxin B_1_ to three unknown fluorescent compounds, designated x_1_, x_2_ and x_3_ and the known aflatoxin M_1_, while the rat was only capable of producing aflatoxin M_1_. The results suggested that metabolites x_1_, x_2_, x_3_ and aflatoxin M_1_ were not part of a major metabolic pathway, but produced independently. These unknown yellowish-green fluorescent compounds did not seem to be conjugated with sulphate or glucuronic acid.

*In vitro* incubations of various mouse liver cell fractions with aflatoxin B_1_ showed that metabolites x_1_, x_2_, x_3_ and aflatoxin M_1_, could only be produced by the microsomal fraction and that NADPH was needed as a co-factor. The differences in aflatoxin metabolism by mice and rats are discussed in relation to the apparent resistance of the mouse to the carcinogenic effects of this toxin.


### A COMPARATIVE STUDY ON AFLATOXIN $B_1$ METABOLISM IN MICE AND RATS

M. STEYN, M. J. PITOUT AND I. F. H. PURCHASE

*From the Division of Toxicology, National Institute for Nutritional Diseases, South African Medical Research Council, Private Bag 380, Pretoria, South Africa*

Received for publication January 14, 1971

**SUMMARY.—*In vivo* metabolic studies on rats and mice revealed a marked difference in the fluorescent compounds produced after ingestion of aflatoxin $B_1$. The mouse converted aflatoxin $B_1$ to three unknown fluorescent compounds, designated $x_1$, $x_2$ and $x_3$ and the known aflatoxin $M_1$, while the rat was only capable of producing aflatoxin $M_1$. The results suggested that metabolites $x_1$, $x_2$, $x_3$ and aflatoxin $M_1$ were not part of a major metabolic pathway, but produced independently. These unknown yellowish-green fluorescent compounds did not seem to be conjugated with sulphate or glucuronic acid.**

***In vitro* incubations of various mouse liver cell fractions with aflatoxin $B_1$ showed that metabolites $x_1$, $x_2$, $x_3$ and aflatoxin $M_1$, could only be produced by the microsomal fraction and that NADPH was needed as a co-factor. The differences in aflatoxin metabolism by mice and rats are discussed in relation to the apparent resistance of the mouse to the carcinogenic effects of this toxin.**

AFLATOXIN $B_1$ is acutely toxic to a number of animal species, including albino mice, which have approximately the same susceptibility to the acute oral effects of aflatoxin $B_1$ (Butler, 1969) as rats (Butler, 1964). In contrast to the rat, the mouse is resistant to the carcinogenic action of aflatoxin $B_1$ (Platonow, 1964; Newberne, 1965; Wogan, 1966).

It is well known that aflatoxin $B_1$ is converted by rats to its hydroxylated derivative, aflatoxin $M_1$, *in vivo* (Patterson and Allcroft, 1970), as well as *in vitro* (Schabort and Steyn, 1969). Portman *et al.* (1968) reported the conversion of aflatoxin $B_1$ to $M_1$ by washed microsomes prepared from mouse liver, but Patterson and Allcroft (1970) and Bassir and Emafo (1970) could not confirm this observation.

This study was undertaken to investigate and establish the difference between mice and rats in their ability to convert aflatoxin $B_1$ into fluorescent metabolites.

### MATERIALS AND METHODS

All chemicals used were of analytical reagent grade. Nicotinamide-adenine dinucleotide phosphate, reduced form (NADPH) was obtained from Boerhinger, West Germany. Protein concentrations were determined as described by Lowry *et al.* (1951). $\beta$-glucoronidase (bovine-liver) and aryl sulphatase (*Helix pomatia*) were obtained from Calbiochem., Switzerland. $^{14}$C-Carboxyl-labelled acetate (specific activity 25 Ci/mole) was obtained from The Radiochemical Centre, Amersham, and used to synthesize $^{14}$C-uniformly labelled aflatoxin $B_1$ according to the method of Adye and Mateles (1964) and purified as described by Steyn (1970).

Albino mice (± 23 g.) obtained from Onderstepoort Veterinary Research Institute and our own Wistar-derived albino rats (weight ± 200 g.) were used in these experiments.

In vivo *studies*

*Aflatoxin $B_1$ metabolism in mice and rats.*—Two male rats (average weight 220 g.), and eight adult male mice (mean weight 23 ± 2 g.) were dosed *per os* with aflatoxin $B_1$ (10 mg./kg.) in dimethylsulphoxide (DMSO) (0·1 ml.) and killed with ether 2 hours later. Their livers, kidneys, stomachs plus intestines and bladders plus urine were excised, weighed and extracted according to Purchase and Steyn (1969), utilising an azeotrope consisting of acetone : chloroform : water (58 : 38 : 4). The concentration of aflatoxin $B_1$ was assayed according to Pons, Robertson and Goldblatt (1966).

*Absorption of aflatoxin $B_1$ from the stomachs of mice.*—Twelve male mice (average weight 23 g.) were each dosed *per os* with aflatoxin $B_1$ (10 mg./kg.) in DMSO (0·1 ml.). Two mice were killed with ether at ½, 1, 2, 4, 6 and 7½ hours after dosing and their stomachs and intestines removed. These organs were assayed for aflatoxin $B_1$ as described above.

*Rate of aflatoxin $B_1$ metabolite formation in mice.*—Twelve male and 12 female mice (average weight 23 ± 2 g.) each received an oral dose of 3·0 mg. aflatoxin $B_1$ in 0·1 ml. DMSO. Two males and two females were killed as before at 20, 40, 70, 100, 150 and 180 minutes after dosing. Their livers, stomachs plus intestines, kidneys and bladders plus urine were removed and treated as above. The relative amounts of the unknown metabolites are expressed in $\mu$g. aflatoxin $B_1$ equivalents as no quantitative standards of the unknown were available.

Two male mice (± 23 g.) each received an oral dose of 3 mg. $^{14}C$ aflatoxin $B_1$ ($30 \times 10^3$ d.p.m./mg. aflatoxin) in 0·1 ml. DMSO and were killed after 100 minutes as described before. Urine was collected from the bladders by means of a 1 ml. syringe (total volume of urine, 0·7 ml.) and chromatographed on 1 mm. thin-layer chromatography (t.l.c.) plates. The fluorescent bands, containing metabolites $x_1$, $x_2$ and aflatoxin $M_1$, were separately collected and the radioactivity measured in a Beckman liquid scintillation system.

In vitro *studies*

*Preparation of liver cell fractions.*—Two adult male mice were decapitated, their livers (total weight 6 g.) removed and homogenized in 18 ml. 0·32M sucrose-3mM $MgCl_2$–0·02M TRIS buffer, pH 7·6, in a Dounce homogenizer. The suspension was centrifuged in an MSE-mistral 2 L centrifuge at 1800 ***g*** for 20 minutes to remove the nuclei, which were discarded. The supernatant was then centrifuged at 9000 ***g*** for 20 minutes in a Spinco L-4 ultracentrifuge, to yield the crude mitochrondrial fraction, which was suspended in 10 ml. 0·02M TRIS buffer, pH 7·4, and kept. The microsomes were obtained by centrifugation of the resultant supernate at 105,000 ***g*** for 60 minutes, washed twice with 10 ml. of the above mentioned buffer and diluted with the same buffer to a protein concentration of 10–15 mg. per ml. The supernatant, which consisted of the soluble cell fraction, was used undiluted.

*Incubation of aflatoxin $B_1$ with different cell fractions.*—The incubation mixtures, with a final volume of 5 ml., were made up as follows:

1. Aflatoxin $B_1$ in methanol or 1,2-propylene glycol (7 mg./ml., 0·4 ml.);
2. 0·02M TRIS buffer, pH 7·4 (3·4 ml.);
3. $MgCl_2$ solution (14·2 g./L, 0·2 ml.);
4. NADPH (74·5 mg./1 ml. TRIS buffer solution): 0·5 ml.;
5. Cell fraction: 0·5 ml.

All incubations were carried out in a shaking water bath at 37° C. in cotton wool plugged test tubes for 30 to 60 minutes. The reactions were terminated by the addition of 10 ml. acetone. Reaction mixtures were transferred quantitatively to suitable flasks by washing with two more 10 ml. portions of acetone.

*Conjugates.*—Enzyme incubations were performed as described by Fishman (1946) and Whitehead *et al.* (1952), after addition of chromatographically pure $x_1$, $x_2$ and $x_3$. These metabolites were also boiled in 6N HCl for 5 minutes (Oser, 1965).

*Chromatographic methods*

All extracts were evaporated to dryness in a rotary evaporator (under reduced pressure) at 45° C., and dissolved in 2 ml. benzene : acetonitrile (95 : 5). T.l.c. plates were prepared from Camag D-5 silica gel (Camag, Switzerland), wet layer thickness 0·5 mm., heated at 100° C. for 2 hours and cooled at room temperature immediately before use. After application of suitable quantities of extracts, as well as a quantitative aflatoxin $B_1$ standard, the chromatograms were developed in an unlined tank containing 100 ml. chloroform : acetone (80 : 20) as the mobile phase.

The following procedures were used to identify aflatoxin $M_1$ in several mouse organs:

1. T.l.c. employing four different mobile phases, namely, chloroform : acetone (80 : 20); chloroform : methanol (95 : 5); trichlorethylene : acetone (10 : 90), and benzene : ethanol : water (46 : 45 : 19).
2. Paper chromatography, as described by Holzapfel *et al.* (1966).
3. Ultra-violet spectrophotometry, employing a Beckman model DK-2A spectrophotometer.

RESULTS

In vivo *experiments*

*Metabolism of aflatoxin $B_1$ by the mouse.*—Fig. 1 illustrates the typical metabolic conversion pattern of aflatoxin $B_1$ by various organs of the mouse and Fig. 2 demonstrates the difference in metabolism of aflatoxin $B_1$ between rat and mouse livers.

From Fig. 1 and 2 it is clear that only the mouse is able to metabolize aflatoxin $B_1$ to three unknown fluorescent metabolites, two major and one minor, designated $x_1$, $x_2$ and $x_3$, respectively. These compounds all fluoresce green-yellowish. The 4th blue fluorescent metabolite with an Rf value between that of $x_1$ and $x_2$ was shown to be chromatographically identical to aflatoxin $M_1$ and to have a similar ultra-violet spectrum. The rate of production of $x_1$, $x_2$ and aflatoxin $M_1$ from combined organ extracts, expressed in $\mu$g. aflatoxin $B_1$ equivalents, is shown in Fig. 3.

From Table I it is evident that metabolites $x_1$, $x_2$ and aflatoxin $M_1$ recovered from the urine of mice dosed with $^{14}C$-aflatoxin $B_1$ are radioactive. Metabolite

Fig. 1

Fig. 2

Fig. 1.—Chromatogram of metabolites extracted from various mouse organs as viewed under ultra-violet light—1: liver; 2: stomach; 3: kidneys; 4: intestines; 5: urine plus bladder.

Fig. 2.—Chromatogram of metabolites extracted from rat and mouse liver 2 hours after administration of aflatoxin $B_1$. 1: rat; 2: qualitative standard containing aflatoxins $B_1$, $M_1$ and $B_{2a}$; 3: mouse.

Fig. 3.—Rate of fluorescent metabolite formation in mice. ● : $x_1$; ⊙ : $M_1$; ○ : $x_2$.

TABLE I.—*Distribution of Radioacitity in Metabolites $x_1$, $x_2$ and Aflatoxin $M_1$ in the Urine of Mice Following an Oral Dose of $^{14}C$-aflatoxin $B_1$*

| Metabolite | d.p.m./ml. urine |
|---|---|
| $x_1$ | 700 |
| $M_1$ | 1410 |
| $x_2$ | 3810 |

$x_3$ was obtained in too low a yield to confirm its relationship to aflatoxin $B_1$ in this way.

No fluorescent substances resembling $B_1$ or its metabolites were observed in extracts from control animals.

The ability of the mouse to absorb consistently more aflatoxin $B_1$ from its stomach than the rat, is illustrated in Fig. 4.

FIG. 4.—The amounts of aflatoxin $B_1$ detected in the stomachs of rats and mice up to 8 hours after dosing. ○ : rat; ● : mouse.

### In vitro *experiments*

*Conversion of aflatoxin $B_1$ by mouse liver cell fractions.*—The ability of the various fractions to metabolize aflatoxin $B_1$ is illustrated in Fig. 5, which distinctly indicates that only the microsomal fraction is capable of producing a variety of fluorescent metabolites. Furthermore, it is also clear that the conversion mechanism, present in the microsomal fraction, needs NADPH (Fig. 5).

*Assay for conjugates.*—Treatment of chromatographically pure $x_1$, $x_2$ and $x_3$ with $\beta$-glucuronidase (bovine liver) aryl sulphatase (Helix pomatia) or 6N HCl showed no effect.

FIG. 5.—The ability of mouse liver cell fractions to convert aflatoxin $B_1$. 1: cell sap; 2: crude mitochondrial fraction; 3: qualitative standard containing aflatoxins $B_1$, $M_1$ and $B_{2a}$; 4: microsomal fraction plus NADPH; 5: microsomal fraction without NADPH.

### DISCUSSION

When a comparison is made between the amount of aflatoxin $B_1$ in rat and mouse stomachs after a single dose, it is clear that the mouse absorbs the toxin more quickly than the rat. As the acute toxicity in the mouse and rat are approximately the same, the mouse must either be more resistant to the effects of the toxin *per se* or it must be able to metabolize (detoxify) the toxin at a greater rate than the rat. The ability of the mouse to produce numerous fluorescent metabolites, and the absence of large quantities of aflatoxin $B_1$ in liver and kidneys indicate that the mouse is capable of metabolizing aflatoxin efficiently.

The absence of carcinogenicity of aflatoxin in mice may also be related to the rapid metabolism. Alternatively, if aflatoxin $B_1$ is converted into an " ultimate " carcinogen by metabolism in the rat, the lack of carcinogenicity could be due to the different metabolic conversion products produced by the mouse. Further studies on the identity and biological activity of aflatoxin metabolites may indicate which of these two suggestions is correct.

The *in vitro* experiments clearly indicate that only the microsomal fraction of mouse liver contains the enzyme(s) necessary for the conversion of aflatoxin $B_1$ to $M_1$ and the other three fluorescent components.

In addition, it is also possible that the cytochrome P-450 component, which is the rate-limiting step in drug conversion of mouse liver microsomes, has a higher affinity for aflatoxin $B_1$ than that of the rat. Since we accept the observation that the mouse is resistant to aflatoxin $B_1$-induced carcinogenicity, the conversion of aflatoxin $B_1$ to the three fluorescent compounds could be used as a monitoring system to study the susceptibility of various animal species, including man, to aflatoxin $B_1$-inducible carcinogenesis. Obviously, much more work has still to be done to validate such a statement.

Although highly unlikely, the possibility existed that the metabolites $x_1$, $x_2$ and aflatoxin $M_1$ were formed in mice through the synergistic action of aflatoxin

$B_1$ and DMSO from some other compound normally present in the animals. This possibility was exluded by using $^{14}C$-aflatoxin $B_1$ (Table I).

Whether aflatoxin $M_1$, metabolites $x_1$, $x_2$ and $x_3$ are minor metabolites or intermediates in a major metabolic pathway in the mouse cannot be deduced from our results. However, the results in Fig. 3 suggest that these metabolites are synthesized independently, since their excreted concentrations are similar at any particular time. Both mouse and rat livers could also metabolize aflatoxin $B_1$ to non-fluorescent compounds which are not perceptible, or to fluorescent metabolites which are not extracted with the acetone : chloroform : water azeotrope. It also seems that $x_1$, $x_2$ and $x_3$ are not glucuronide or sulphate conjugates.

The results of this study clearly demonstrate that the mouse can hydroxylate aflatoxin $B_1$ to aflatoxin $M_1$. This is in agreement with the results from Portman *et al.* (1968) who suggested that mouse liver microsomes hydroxylate aflatoxin $B_1$ (forming $M_1$) faster than those of rat liver. However, these authors did not mention the presence of other fluorescent components. Although Bassir and Emafo (1970) failed to demonstrate $M_1$ production by mouse liver, they did describe the presence of a green-yellow fluorescent substance. Whether their unknown fluorescent compound corresponds with our $x_1$, $x_2$ or $x_3$ is at present unknown. These differences could be due to different strains of mice or to different extraction techniques used by the various authors.

The authors wish to thank Mr. N. P. Ferreira of the Microbiology Research Group, C.S.I.R., Pretoria, Republic of South Africa, for the production of $^{14}C$-aflatoxin $B_1$.

### REFERENCES

Adye, J. and Mateles, R. I.—(1964) *Biochem. biophys. Acta*, **86**, 418.

Bassir, O. and Emafo, P. O.—(1970) *Biochem. Pharmac.*, **19**, 1681.

Butler, W. H.—(1964) *Br. J. Cancer*, **18**, 756.—(1969) *In* ' Aflatoxin, Scientific Background, Control and Implications ', by L. A. Goldblatt. New York and London (Academic Press), p. 234.

Fishman, W.—(1946) *J. biol. Chem.*, **166**, 757.

Holzapfel, C. W., Steyn, P. S. and Purchase, I. F. H.—(1966) *Tetrahedron Lett.*, **25**, 2799.

Lowry, O. H., Rosebrough, N. J., Farr, A. L. and Randall, R. J.—(1951) *J. biol. Chem.*, **193**, 265.

Newberne, P. M.—(1965) *In*: ' Mycotoxins in Foodstuffs ' edited by G. N. Wogan. Cambridge, Mass. (M.I.T. Press), p. 187.

Oser, B. L., editor (1965) ' *Hawk's physiological chemistry* '. New York (McGraw Hill), p. 1181.

Patterson, D. S. P. and Allcroft, R.—(1970) *Fd Cosmet. Toxic.*, **8**, 43.

Platonow, N.—(1964) *Vet. Rec.*, **76**, 589.

Pons, W. A. Jr., Robertson, J. A. and Goldblatt, L. A.—(1966) *J. Am. Oil Chem. Soc.*, **43**, 665.

Portman, R. S., Plowman, K. M. and Campbell, T. C.—(1968) *Biochem. biophys. Res. Commun.*, **33**, 711.

Purchase, I. F. H. and Steyn, M.—(1969) *Br. J. Cancer*, **23**, 800.

Schabort, J. C. and Steyn, M.—(1969) *Biochem. Pharmac.*, **18**, 2241.

Steyn, M.—(1970) *Ass. Off. Anal. Chem.*, **53**, 619.

Whitehead, J. E. M., Morrison, A. R. and Young, L.—(1952) *Biochem. J.*, **51**, 585.

Wogan, G. N.—(1966) *Bact. Rev.*, **30**, 460.